# TRIM56 Promotes Antiviral Responses Downstream of TLR4

**DOI:** 10.3390/v18070792

**Published:** 2026-07-19

**Authors:** Xiaohan Tong, Nan L. Li, Darong Yang, Benjamin M. Liu, Zhuoyuan Alex Li, Kui Li

**Affiliations:** 1 Department of Microbiology, Immunology and Biochemistry, University of Tennessee Health Science Center, 858 Madison Avenue, Memphis, TN 38163, USA; 2 College of Medicine, University of Tennessee Health Science Center, Memphis, TN 38163, USA

**Keywords:** TRIM56, TLR4, IRF3, interferon-stimulated genes, antiviral response, TRIF

## Abstract

The ubiquitin ligase protein tripartite-motif containing 56 (TRIM56) positively regulates Toll-like receptor-3 (TLR3) signaling by forming a complex with Toll-Interleukin-1 receptor domain-containing adapter protein inducing interferon (IFN)-beta (TRIF), independent of its E3 ligase activity. Whether TRIM56 modulates other TLR pathways in innate antiviral immunity, however, is unclear. Herein, we show ectopic expression of TRIM56 augments activation of IFN regulatory factor-3 (IRF3)-dependent promoters following stimulation by lipopolysaccharide (LPS) in HEK293-TLR4-MD2-CD14 cells while leaving activation of NF-κB-dependent promoter unaffected, suggesting TRIM56 specifically promotes immune signaling through the TLR4-TRIF axis but not the MYD88 arm downstream of this TLR. Confirming its impact on endogenous antiviral responses in immune sentinel cells naturally harboring the TLR4 pathway, we demonstrated enforced expression of TRIM56 enhanced LPS-induced expression of IFN-beta and IFN-stimulated genes (ISGs) and establishment of an antiviral state in bone marrow-derived macrophages. Importantly, depletion of endogenous TRIM56 impaired LPS-induced antiviral gene expression and cellular antiviral defense. Altogether, these data add to understanding of the role of TRIM56 in TLR-mediated innate immune responses. Given that TRIM56 is an ISG and that many immune adjuvants and some viral proteins activate TLR4, the findings of this study could have implications for designing immunotherapies, especially those against viral infections.

## 1. Introduction

Pattern recognition receptors (PRRs) such as the membrane-bound Toll-like receptors (TLRs) play important roles in initiating mammalian innate immune responses against microbial infections, including those by viruses. Upon engagement by their cognate ligands early after infection, PRRs recruit specific adaptor proteins, eliciting intracellular signaling pathways that converge on the activation of latent transcription factors, interferon (IFN)-regulatory factor-3 (IRF3) and NF-κB, which coordinately induce the expression of IFNs, IFN-stimulated genes (ISGs) and inflammatory mediators. Antiviral ISGs suppress viral replication in infected cells and establish an antiviral state in neighboring uninfected cells, buying time for the development of antigen-specific adaptive immunity. Additionally, the latter is shaped by secreted IFNs and inflammatory cytokines, which regulate the homing and activation of various immune cells [1,2]. Of the TLRs involved in orchestrating innate antiviral immunity, two enlist and depend on the Toll-Interleukin-1 receptor domain-containing adapter inducing IFN-beta (TRIF)—TLR3 and TLR4—to activate IRF3-dependent antiviral response, with TLR3 recognizing viral double-stranded RNA (dsRNA) while TLR4 sensing lipopolysaccharide (LPS) and some viral proteins [1].

A member of the tripartite-motif containing (TRIM) family of E3 ubiquitin ligases, TRIM56 has garnered increasing attention for its direct antiviral effects against various RNA viruses as well as its roles in regulating immune signaling [2,3,4,5,6,7,8,9]. We have shown that TRIM56 promotes TLR3-mediated innate immune responses following stimulation by extracellular dsRNA or during hepatitis C virus infection [6,7,8]. Interestingly, TRIM56 executes this role in a non-canonical, E3 ligase-independent manner that hinges on its C-terminal portion forming a complex with TRIF, the TLR3 adaptor [6,7]. However, whether TRIM56 modulates innate immune signaling downstream of other TLRs, is unclear. In this study, we sought to determine the impact of TRIM56 on host responses downstream of TLR4, which requires TRIF to elicit IRF3-dependent expression of antiviral genes whereas critically depends on myeloid differentiation primary response 88 (MYD88) to activate NF-κB [10].

## 2. Methods

### 2.1. Cells and TLR Ligands

HEK293 cells stably expressing human TLR4, MD2 and CD14 genes (HEK293-hTLR4-MD2-CD14, InvivoGen, San Diego, CA, USA), C57BL/6J wild-type mouse bone marrow-derived macrophage (BMDM) cell line (BEI resources, Manassas, VA, USA; referred to as B6Mφ), and Vero cells (ATCC, Manassas, VA, USA) were maintained in Dulbecco’s Modified Eagle Medium supplemented with 10% fetal bovine serum, 100 U/mL of penicillin and 100 μg/mL streptomycin at 37 °C with 5% CO_2_ in air atmosphere. We created a B6Mφ pool that stably expresses C-terminally FLAG-tagged human TRIM56 by retroviral gene transfer following described procedure [9].

Poly(I:C12U) (AA Blocks, San Diego, CA, USA) and LPS (Invivogen, San Diego, CA, USA) were added to culture medium at a final concentration of 30 µg/mL and 1 µg/mL, respectively, for stimulation of cells for different times as specified.

### 2.2. Plasmids

pcDNA3.1-TRIM56-V5His6 and its empty vector control pcDNA3.1-V5His [9], reporter constructs containing repeated PRDI (p55C1B-Luc) [11] and repeated PRDII (PRDII-Luc) [12] from the IFN-beta promoter, have been described. hZAP/IRF-E-Luc was constructed such that the ~0.3 kb long, distal region of human zinc-finger antiviral protein (hZAP) promoter encompassing the five IRF elements (IRF-Es) controls expression of the firefly luciferase reporter gene. It was engineered from the hZAP(-2486)-GL3 reporter construct [13] by Quikchange PCR mutagenesis deleting a ~2.1 kb long, internal region of hZAP promoter and joining the distal IRF-Es and the TATA box. The mutagenesis primers used were 5′-CCTCTGTTGCCTTTGCTAGCGTCACACGCCTCAG-3′ (forward) and 5′-CTGAGGCGTGTGACGCTAGCAAAGGCAACAGAGG-3′ (reverse), with the introduced NheI site underlined. pRL-TK (Promega, Madison, WI, USA) was used for normalization of transfection efficiency.

### 2.3. RNA Interference (RNAi)

To deplete endogenous mouse TRIM56, B6Mφ cells were transduced with a mix of two pLKO.1-based lentiviral vectors, each carrying a mouse Trim56-specific shRNA designed using algorithms that not only predict knockdown performance but also reduce off-target gene knockdown events (Open Biosystems, Huntsville, AL, USA). The target sequences were CGCCTTTAAGACCAACTTCTT (TRCN0000037385) and CCATGTCTTTAGGGTCAGGTT (TRCN0000037388). Following selection in medium containing 8 µg/mL of puromycin, surviving cell colonies were pooled and used for analyses.

### 2.4. Reporter Gene Assay

The activities of IRF3-dependent PRDI and hZAP-IRF-E promoters and that of NF-κB-dependent PRDII promoter in transfected cells with and without LPS stimulation were determined using dual-luciferase assay as described [14,15].

### 2.5. RNA Analyses

Total RNA extraction, cDNA synthesis, and quantitative reverse transcription-PCR (qRT-PCR) using SYBR green technology were conducted as described [8,15,16]. The following gene-specific primers were used: Ifnb1, 5′-TCCGAGCAGAGATCTTCAGGAA-3′ (forward) and 5′-TGCAACCACCACTCATTCTGAG-3′ (reverse); Isg15, 5′-AAGAAGCAGATTGCCCAGAA-3′ (forward) and 5′-TCGCTGCAGTTCTGTACCAC-3′ (reverse); Mda5 (a.k.a., Ifih1), 5′-ACAGAGGCCTGGAACGTAGA-3′ (forward) and 5′-TTCATCGAAGCAGCTGACAC-3′ (reverse); Trim56, 5′-CAAGGGGACGATAGAACCAA-3′ (forward) and 5′-CCTGTTACCCCTTTCGATGA-3′ (reverse). The primers for Ifit1, Ifit2 and Ifit3 have been described [17]. Relative gene expression was calculated using the 2^−ΔΔCt^ method, where ΔCt = Ct(target gene) − Ct(28S rRNA) and ΔΔCt = ΔCt(sample) − ΔCt(control). Gene expression levels were normalized to 28S rRNA and expressed as fold change relative to mock-treated control.

### 2.6. Immunoblotting

Cell lysates were prepared and subjected to SDS-PAGE and subsequent immunoblotting as described [8,14,15]. The following primary antibodies were used for immunodetection: rabbit anti-MDA5 (Proteintech, Rosemont, IL, USA), mouse anti-ISG15 (Santa Cruz, Dallas, TX, USA), rabbit anti-IFIT3 (Invitrogen, Carlsbad, CA, USA), rabbit anti-TRIM56 S4091 [6], and mouse anti-beta-ACTIN (ABclonal, Woburn, MA, USA). Protein bands were visualized following incubation with appropriate IRDye^®^-labeled secondary antibodies and their signal intensity quantified using Image Studio Lite (LI-COR Biosciences, Lincoln, NE, USA) and normalized to that of beta-ACTIN.

### 2.7. VSV-Luc-Based Antiviral Activity Assay

Cells with and without pre-stimulation by LPS for 8 h were challenged by a recombinant vesicular stomatitis virus expressing firefly luciferase (VSV-Luc) at MOI = 0.1 for 8 h, followed by cell lysis and luciferase assay as described [8,18].

### 2.8. Fifty Percent Tissue Culture Infectious Dose (TCID50) Assay of Viral Titers

Progeny virus titers in culture supernatant samples were determined by a cytopathic effect (CPE)-based TCID50 assay on Vero cells seeded in 96-well plates as described [17] and expressed as TCID50/mL.

### 2.9. Statistical Analyses

Data are presented as mean ± SD. Statistical analyses were performed using GraphPad Prism software (Version 8). Comparisons between two experimental groups were performed using Student’s *t*-test. For experiments involving multiple groups, data were analyzed by one-way ANOVA followed by Sidak’s multiple comparisons test for prespecified pairwise comparisons. For the data from time-course experiments, each cell type/time point combination was treated as an individual experimental group, and comparisons were made between the two cell groups at the corresponding time points. A *p* value of <0.05 was considered statistically significant.

## 3. Results and Discussion

### 3.1. TRIM56 Augments LPS-Induced Activation of IRF3-Dependent Promoters in HEK293-hTLR4-MD2-CD14 Cells

**Figure 1:**
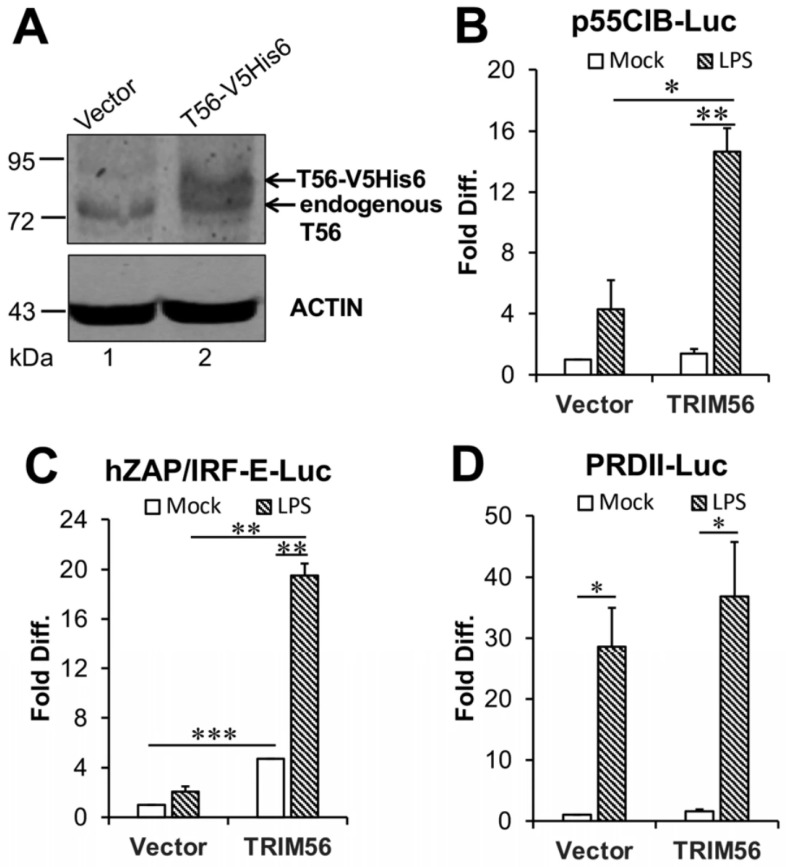
Impact of ectopic expression of TRIM56 on LPS-induced activation of IRF3- and NF-kB-dependent promoters in HEK293-TLR4-MD2-CD14 cells. (**A**) Immunoblot analysis of TRIM56 expression in HEK293-hTLR4-MD2-CD14 cells transfected with empty vector (pcDNA3.1-V5His6) or TRIM56-V5His6 expression vector. The endogenous TRIM56 and ectopically expressed TRIM56-V5His6 proteins are marked by arrows. Activation of IRF-3-dependent PRDI promoter (p55CIB-Luc, (**B**)) and hZAP-IRF-E promoter (**C**), or NF-κB-dependent PRDII promoter (**D**) in HEK293-TLR4-MD2-CD14 cells transiently expressing TRIM56 or an empty control vector, and mock-stimulated (empty bars) or stimulated by LPS (hatched bars) for 16 h. Data are expressed as mean ± SD from two independent experiments. *, **, and *** denote *p* < 0.05, *p* < 0.01, and *p* < 0.001, respectively (Student’s *t*-test).

Apart from being the sole adaptor protein for TLR3, TRIF is also employed by TLR4 and critical for induction of IRF3-dependent genes downstream of this pathway [10]. We hypothesized that the physical interaction between TRIM56 and TRIF [6,7] would influence TRIF-dependent signaling downstream of TLR4. To test this, HEK293-hTLR4-MD2-CD14 cells were transfected with a C-terminally V5-His6 tagged, TRIM56-encoding vector or its empty vector as a control. Immunoblot analysis confirmed the expression of ectopic TRIM56-V5-His6 in the former group, at levels comparable to endogenous TRIM56 (Figure 1A). It is worth noting that human TRIM56 protein is ubiquitously expressed across different tissues albeit at varying levels [9] and TRIM56 RNA expression is present in a vast majority of human cell lines (Table S1), including HeLa, HEK293, THP1, Huh7, and SVGA, that express readily detectable TRIM56 protein [4,5,6,7,8,9,19]. We set out to determine the impact of transient expression of TRIM56 on LPS-induced activation of IRF3- and NF-κB-dependent promoters in HEK293-hTLR4-MD2-CD14 cells. In cells transfected with the empty vector, LPS stimulation weakly activated the 55C1B promoter (by 2.9-fold), which is driven by the IRF3-dependent PRDI motif from the IFN-β promoter. In comparison, LPS had a significantly greater effect in cells ectopically expressing TRIM56, stimulating the promoter by 9.8-fold (Figure 1B). The same can be said when we examined LPS-induced hZAP/IRF-E promoter (Figure 1C), which comprises a DNA fragment from human ZAP promoter encompassing five IRF elements that specifically bind activated IRF3 during viral infection [13]. Specifically, LPS stimulated hZAP/IRF-E promoter by 1.7-fold and 4.3-fold, in empty vector- and TRIM56 expression vector-transfected cells, respectively, compared with their own mock-stimulated controls. Taken together, these data show that TRIM56 augments transcription from IRF3-dependent promoters upon engagement of the TLR4 pathway, which is known to be dependent on TRIF.

Notably, TRIM56 expression alone without LPS stimulation also significantly upregulated the hZAP/IRF-E promoter (by 4.7-fold) (Figure 1C). This effect was specific to the hZAP/IRF-E reporter, as TRIM56 expression *per se* did not activate the 55C1B promoter (Figure 1B). It will be interesting to determine in future studies how TRIM56 overexpression may increase ZAP levels and whether ZAP, an RNA-binding protein that promotes viral RNA degradation [20], has any role in TRIM56-mediated direct antiviral effects against specific viruses [4,5,9,19].

By contrast, we observed robust activation of the NF-κB-dependent PRDII promoter by LPS stimulation in cells with and without TRIM56 ectopic expression, with no significant difference between the two (Figure 1D). Since NF-κB activation via the TLR4 pathway are predominantly MYD88-dependent [10], the lack of an effect of TRIM56 on LPS-induced NF-κB activation suggests that heightened TRIM56 expression does not promote signaling via MYD88. Consistent with this conclusion, our recent study has shown TRIM56 overexpression has no demonstrable effect on activation of the PRDII promoter by IL-1β [6], which is also MYD88-dependent.

### 3.2. TRIM56 Enhances LPS-Induced Expression of Ifnb1 and Antiviral ISGs in BMDMs

**Figure 2:**
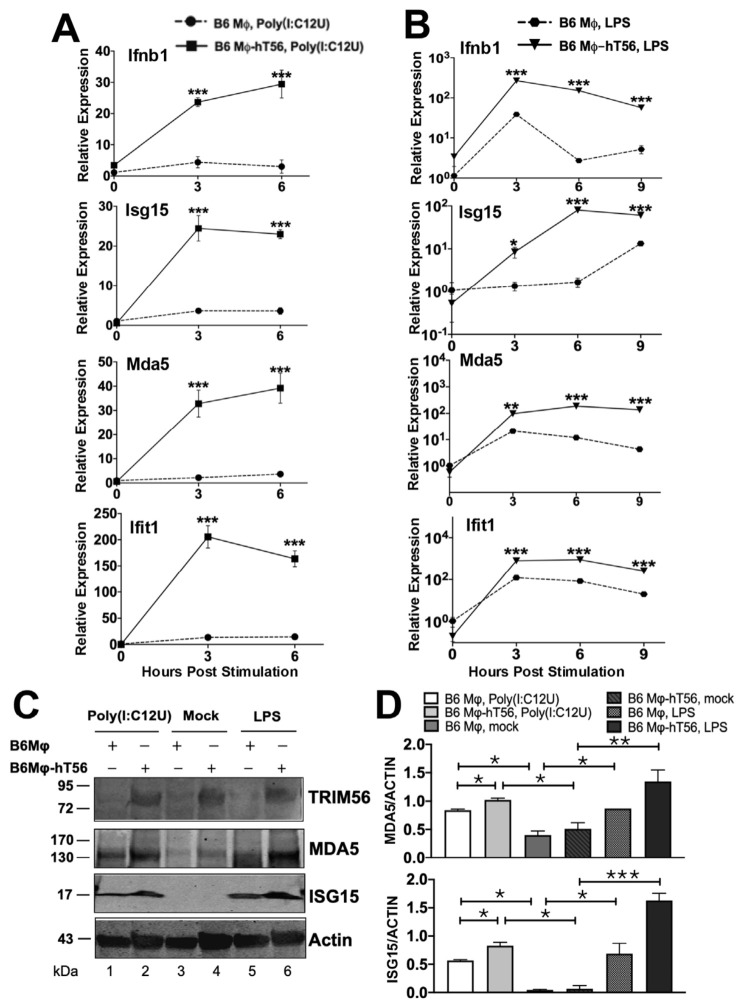
Enhanced antiviral gene expression in TRIM56-expressing bone marrow-derived macrophages following LPS stimulation. (**A**,**B**) qRT-PCR analyses of the abundance of indicated gene transcripts in B6Mϕ with or without TRIM56-FLAG expression at indicated times post stimulation by Poly(I:C12U) (**A**) or LPS (**B**). *, **, and *** denote *p* < 0.05, *p* < 0.01, and *p* < 0.001, respectively (one-way ANOVA followed by Sidak’s multiple comparisons test). (**C**) Immunoblotting of TRIM56, MDA5, ISG15, and ACTIN protein levels in B6Mϕ with or without TRIM56-FLAG expression, mock-stimulated or stimulated by Poly(I:C12U) or LPS for 8 h. Quantification of the immunoblotting data by densitometry analysis (normalized to ACTIN) is presented as bar graphs in (**D**). Data are from two independent experiments. Error bars represent SD. *, **, and *** denote *p* < 0.05, *p* < 0.01, and *p* < 0.001, respectively (Student’s *t*-test).

To confirm the effect of TRIM56 in immune sentinel cells that naturally harbor the TLR4 pathway, we conducted further experiments in B6Mφ cells with and without TRIM56 expression delivered by retroviral gene transfer. qRT-PCR demonstrated that when stimulated by Poly(I:C12U), a dsRNA surrogate specifically engaging the TLR3 pathway without activating the cytosolic retinoic acid-inducible gene I (RIG-I)-like receptors (RLRs, RIG-I and MDA5) [21,22,23], B6Mφ expressing TRIM56 (B6Mφ-hT56) had significantly greater induction of Ifnb1 and several representative antiviral ISGs—Isg15, Mda5, and Ifit1—than B6Mφ without (Figure 2A). These data are consistent with our previous reports that TRIM56 promotes TLR3 signaling in non-immune cells HeLa, HT1080, HEK293-TLR3, and Huh7.5-TLR3 [6,7,8], and suggest this conclusion also holds in an immune cell type. Time-course analyses of the expression of antiviral genes following LPS stimulation revealed a similar phenotype, with B6Mφ-hT56 cells exhibiting ~1-log or greater induction of Ifnb1, Isg15, Mda5, and Ifit1 than in control B6Mφ (Figure 2B). Immunoblot analyses demonstrated heightened induction of MDA5 and ISG15 proteins in B6Mφ-hT56 than in control B6Mφ following stimulation by either Poly(I:C12U) (Figure 2C,D, compare lanes 2 vs. 1) or LPS (compare lanes 6 vs. 5). In aggregate, these data ascertain our earlier reporter gene assay data from HEK293-hTLR4-MD2-CD14 cells (Figure 1) and illustrate that TRIM56 promotes the induction of antiviral innate immune genes via TLR3 or TLR4 signaling in murine BMDMs. Along this line, we have previously demonstrated that mouse TRIM56, which shares 81% overall sequence identity with (albeit 21 amino acids shorter than) its human counterpart, enhances TLR3 signaling in human cells [6]. Of note, the C-terminal NHL-like domain of TRIM56 critical for its interaction with TRIF is highly conserved between the two species, sharing ~89% sequence identity and both predicted to fold into a six-bladed beta-propeller [3,4]. In addition, the three phospho-acceptor Serine residues in a C-terminal intrinsically disordered region of human TRIM56−Ser-471, Ser-475, and Ser-710−critical for augmenting TLR3 signaling, are also conserved in mouse TRIM56 (Ser-450, Ser-454, and Ser-689, respectively) [6]. Although remaining to be determined, these traits could help explain why human and mouse TRIM56 proteins are interchangeable for their function of positively regulating TRIF-dependent signaling, at least in cell culture models.

### 3.3. Depletion of Endogenous TRIM56 Impairs LPS-Induced Expression of Antiviral Genes in BMDMs

**Figure 3:**
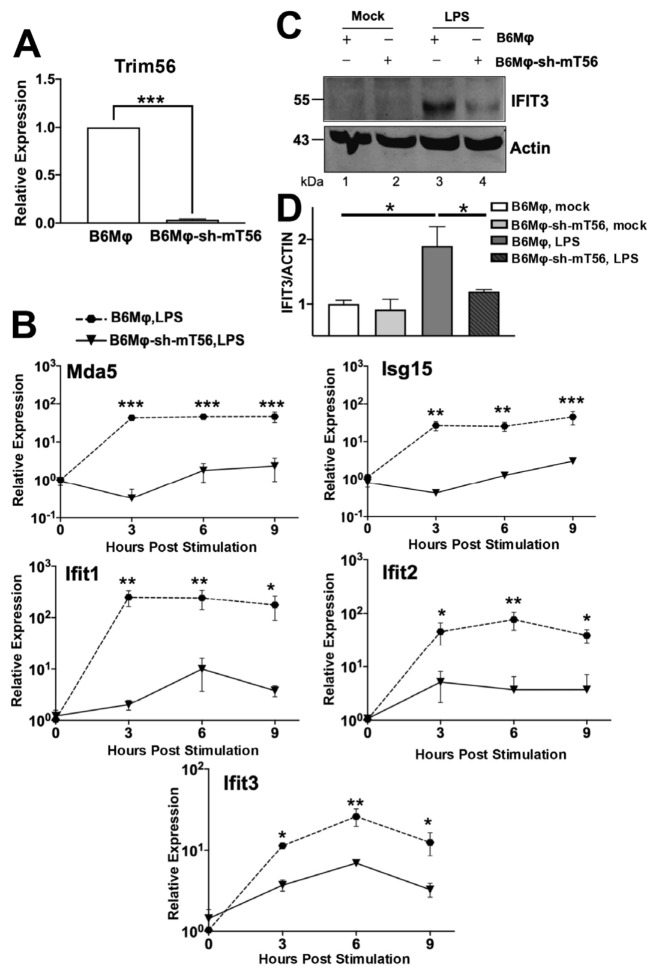
Impaired antiviral gene expression following LPS stimulation in Trim56-depleted BMDMs. (**A**) Relative Trim56 mRNA levels determined by qRT-PCR in control B6Mϕ and B6Mϕ-sh-mT56 with stable knockdown of Trim56. Data are presented as mean ± SD from two independent experiments. *** *p* < 0.001 (Student’s *t*-test). (**B**) qRT-PCR analyses of the abundance of indicated gene transcripts in control B6Mφ and B6φ-sh-mT56 at indicated times post stimulation by LPS. *, **, and *** denote *p* < 0.05, *p* < 0.01, and *p* < 0.001, respectively (one-way ANOVA followed by Sidak’s multiple comparisons test). (**C**) Immunoblotting of IFIT3 and ACTIN protein levels in control B6Mϕ and B6ϕ-sh-mT56, mock-stimulated or stimulated by LPS for 8 h. Densitometric quantification of the immunoblotting data from two independent experiments, normalized to ACTIN, is shown in (**D**). * denotes *p* < 0.05 (Student’s *t*-test).

To determine the contribution of endogenous TRIM56 to TLR4-dependent antiviral responses, we depleted mouse TRIM56 in B6Mφ cells by shRNA-mediated knockdown (referred to as B6Mφ-sh-mT56). Because none of the TRIM56 antibodies we had access to could detect endogenous mouse TRIM56 protein, qRT-PCR was performed and it verified the highly efficient knockdown of Trim56 mRNA (Figure 3A). We next compared antiviral gene induction following LPS stimulation in control B6Mφ and B6Mφ-sh-mT56 cells. Time-course qRT-PCR analyses revealed the LPS induction of multiple, representative antiviral genes, including Mda5, Isg15, Ifit1, Ifit2, and Ifit3, was all significantly reduced in B6Mφ-sh-mT56 cells (Figure 3B). Immunoblotting analysis validated the substantially weakened induction of IFIT3 protein in cells depleted for mouse Trim56 (Figure 3C,D). Taken together, these results support that TRIM56 expressed at physiologic levels is required for optimal induction of antiviral genes downstream of TLR4 signaling in BMDMs.

### 3.4. Ectopic Expression of TRIM56 Potentiates, While Its Depletion Impairs, LPS-Induced Establishment of an Antiviral State in BMDMs

**Figure 4:**
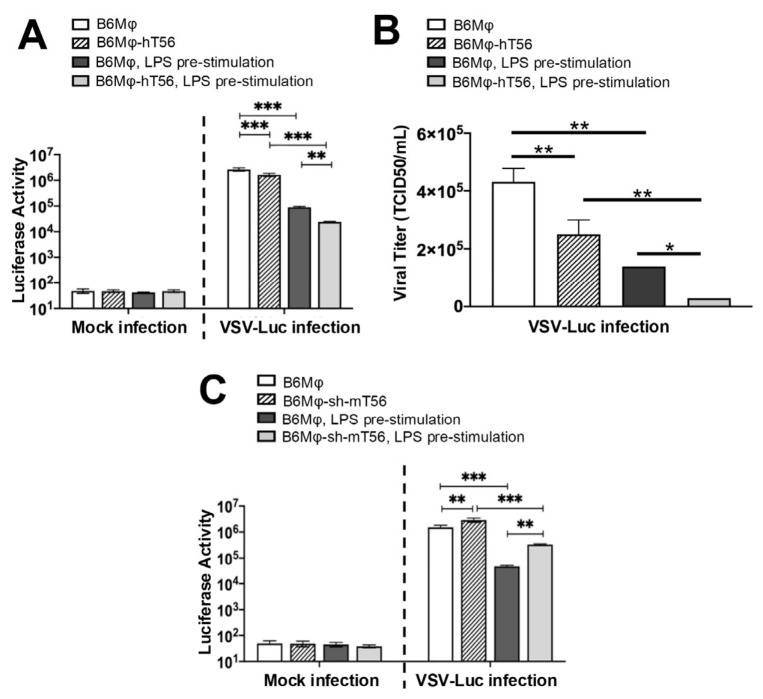
Ectopic expression of TRIM56 potentiates, while its depletion impairs, the establishment of an antiviral state by LPS pre-stimulation against VSV-Luc challenge. (**A**) B6Mφ cells with and without stable expression of human TRIM56 were mock-stimulated or stimulated by 1 μg/mL LPS for 8 h, followed by mock infection or infection by VSV-Luc (MOI = 0.1). Eight hours later, cells were lysed for firefly luciferase assay. (**B**) Culture supernatants colleted under the experimental conditions described in (**A**) were subjected to TCID50 assay on Vero cells to measure progeny virus titers. (**C**) B6Mφ cells with and without shRNA-mediated depletion of endogenous Trim56 were mock-stimulated or stimulated by 1 μg/mL LPS for 8 h, followed by mock infection or infection by VSV-Luc (MOI = 0.1). Eight hours later, cells were lysed for firefly luciferase assay. Data are presented as mean ± SD from two independent experiments. *, ** and *** denote *p* < 0.05, *p* < 0.01 and *p* < 0.001, respectively (one-way ANOVA followed by Sidak’s multiple comparisons test).

We next sought to directly gauge the impact of TRIM56 on antiviral protection conferred by TLR4 signaling. B6Mφ and B6Mφ-hT56 cells were pre-stimulated by LPS or mock-stimulated, followed by challenge with VSV-Luc that expresses luciferase reporter as a readout for viral replication. The VSV-Luc challenge assay has demonstrated excellent correlation of intracellular luciferase expression with VSV RNA replication and progeny virus yields [17]. As shown in Figure 4A, enforced expression of TRIM56 *per se* marginally reduced VSV-Luc replication (compare B6Mφ and B6Mφ-hT56, by 1.6-fold), in keeping with our previous reports that TRIM56 alone has no significant antiviral activity against VSV in MDBK and HeLa cells [4,7,8,9]. Pre-stimulation by LPS induced a potent antiviral state in both control B6Mφ and B6Mφ-hT56 cells, curtailing viral replication by 29.6-fold and 68.0-fold, respectively, as compared with their un-stimulated counterparts. Notably, LPS pre-stimulation was significantly more effective in B6Mφ-hT56 than in control B6Mφ. Consistent with the luciferase reporter data, progeny virus titers in culture supernatants were 1.7-fold lower in mock-stimulated B6Mφ-hT56 cells than those in mock-stimulated B6Mφ cells. While LPS pre-stimulation significantly reduced virus yields in both cell populations, its effect was greater in B6Mφ-hT56 cells, with progeny virus titers 4.9-fold lower than those in B6Mφ cells (Figure 4B). Together, these data lend direct support to the notion that increased TRIM56 expression enables heightened cellular antiviral protection following engagement of the TLR4 pathway. Whether this is strictly dependent on type I IFN response or other LPS-induced pathways may also contribute will need further study.

Lastly, we conducted the VSV-Luc challenge experiments in B6Mφ with and without stable depletion of Trim56. As shown in Figure 4C, B6Mφ-sh-mT56 cells were slightly more permissive to viral replication than control B6Mφ, allowing 1.9-fold higher luciferase expression than the latter. LPS pre-stimulation conferred strong antiviral protection against VSV-Luc challenge, but its efficacy was significantly reduced in Trim56-depleted cells. Specifically, while pre-stimulation by LPS decreased viral replication by 32.9-fold in control B6Mφ, it did so by merely 8.7-fold in B6Mφ-sh-mT56 cells. These data ascertain the conclusion that the endogenous level of TRIM56 plays a critical part in TLR4-mediated establishment of a cellular antiviral state.

**Figure 5:**
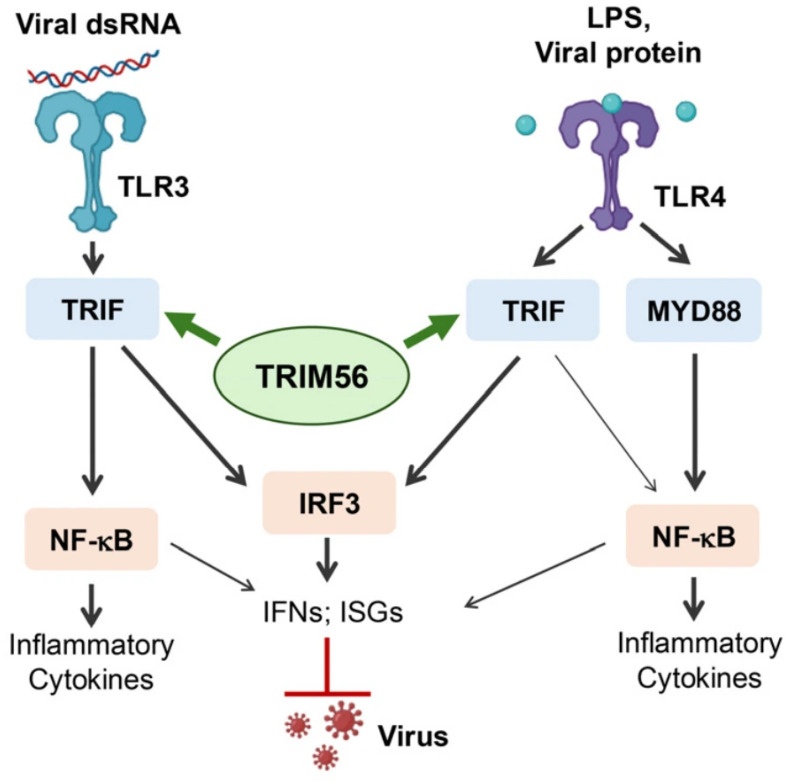
Model of TRIM56-mediated positive regulation of antiviral responses downstream of TLR3 and TLR4. By forming a complex with the adaptor protein TRIF, TRIM56 promotes the induction of IFN genes and antiviral ISGs following engagement of TLR3 by viral dsRNA or engagement of TLR4 by LPS or certain viral proteins, thereby heightening the establishment of a cellular antiviral state that inhibits virus multiplication.

In summary, this study demonstrates that TRIM56 promotes the induction of antiviral responses downstream of TLR4, adding to knowledge of the involvement of TRIM56 in TLR signaling and aiding a fuller understanding of the roles TRIM56 plays in innate immunity (Figure 5). Given that TRIM56 is an ISG upregulated during viral infection [9] and that many immune adjuvants and some viral proteins activate TLR4, TRIM56 could present a node for developing immunotherapies, especially those against viral infections. Future studies in TRIM56-deficient primary macrophages and animal models are warranted to probe this aspect and to investigate the physiological roles of TRIM56 in TLR signaling and other immune mechanisms.

## Data Availability

All the data are contained within the article and Supplementary Materials. Raw data can be made available upon request.

## Supplementary Materials

The following supporting information can be downloaded at: https://www.mdpi.com/article/10.3390/v18070792/s1, Table S1: TRIM56 RNA expression (nTPM) in representative human cell lines [24].

## Abbreviations

The following abbreviations are used in this manuscript:

TRIM56	Tripartite-motif containing 56
TLR	Toll-like receptor
IFN	Interferon
TRIF	Toll-Interleukin-1 receptor domain containing adapter protein inducing IFN beta
IRF3	Interferon regulatory factor-3
MYD88	Myeloid differentiation primary response 88
PRR	Pattern recognition receptor
ISG	Interferon-stimulated gene
IRF-E	IRF element
dsRNA	Double-stranded RNA
BMDM	Bone marrow-derived macrophage
ZAP	Zinc-finger antiviral protein
TCID50	Fifty percent tissue culture infectious dose
RIG-I	retinoic acid-inducible gene I
RLR	RIG-I-like receptor
VSV	Vesicular stomatitis virus
