# Supplementary material for: TRIM56 Promotes Antiviral Responses Downstream of TLR4
Source: Viruses. 2026 Jul 19;18(7):792. doi: 10.3390/v18070792 (PMC13431572; doi:10.3390/v18070792)
Supplement: Supplementary file 1 [file viruses-18-00792-s001.zip › viruses-4438039-supplementary.pdf]

Table S1. TRIM56 RNA expression (nTPM) in representative human cell lines.

Cell line	Cell/Cancer type	Tissue origin	RNA expression (nTPM)
A-431	Skin carcinoma	Epidermis	15.9
A-549	Lung carcinoma	Lung	16.8
BEWO	Choriocarcinoma	Placenta	9.6
BJ	Fibroblast	Skin	12.7
BJAB	Burkitt lymphoma	B lymphocyte	11.1
CACO2	Colorectal carcinoma	Colon	11.1
Calu-3	Lung adenocarcinoma	Lung	10.7
CAPAN2	Pancreatic adenocarcinoma	Pancreas	30.4
Caki-2	Renal cell carcinoma	Kidney	20.2
Daudi	Burkitt lymphoma	B lymphocyte	22.9
DU145	Prostate carcinoma	Prostate	14.5
EFO-21	Ovarian carcinoma	Ovary	17.3
FU97	Gastric carcinoma	Gastric	11.8
HaCaT	Keratinocyte	Skin	17.4
HCT-116	Colorectal carcinoma	Colon	8.9
HEC-1-B	Endometrial adenocarcinoma	Endometrium	9.4
HEK293	Embryonic kidney	Kidney	5.1
HeLa	Cervical carcinoma	Cervix	20.7
HEL	Erythroleukemia	Bone marrow	12.0
HepG2	Hepatocellular carcinoma	Liver	9.0
HL-60	Promyelocytic leukemia	Blood	21.2
HT-1080	Fibrosarcoma	Connective tissue	15.0
HT-29	Colorectal adenocarcinoma	Colon	25.2
Huh-7	Hepatocellular carcinoma	Liver	15.9
K562	Chronic myelogenous leukemia	Blood	22.2
LNCaP	Prostate adenocarcinoma	Prostate	12.3

MCF7	Breast adenocarcinoma	Breast	19.5
MDA-MB-231	Breast adenocarcinoma	Breast	20.8
NCI-H1650	Lung adenocarcinoma	Lung	16.6
NTERA2	Embryonal carcinoma	Testis	10.6
PANC-1	Pancreatic carcinoma	Pancreas	16.0
PLC/PRF/5	Hepatocellular carcinoma	Liver	11.2
RD	Embryonal rhabdomyosarcoma	Muscle	24.8
Rh30	Rhabdomyosarcoma	Muscle	13.3
RT-4	Bladder carcinoma	Bladder	18.1
SH-SY5Y	Neuroblastoma	Nervous tissue	0.3
SK-MEL-30	Melanoma	Skin	12.0
SK-OV-3	Ovarian adenocarcinoma	Ovary	5.3
SuSa	Testicular teratocarcinoma	Testis	10.1
T-47d	Breast ductal carcinoma	Breast	7.9
THP-1	Monocytic leukemia	Blood	20.1
U-138MG	Glioblastoma	Brain	28.5
U-251MG	Glioblastoma	Brain	17.7
U2OS	Osteosarcoma	Bone	27.0
U-698-M	Diffuse large B-cell lymphoma	Lymph node	17.3
U-87-MG	Glioblastoma	Brain	16.1
U-937	Histiocytic lymphoma	Blood	24.0

Data source[1]**:**
Human Protein Atlas (HPA) Cell Line Atlas (proteinatlas.org, accessed on June 18, 2026). TRIM56 RNA expression values are reported as normalized transcripts per million (nTPM).

Primary reference:

1. Jin, H.; Zhang, C.; Zwahlen, M.; von Feilitzen, K.; Karlsson, M.; Shi, M.; Yuan, M.; Song, X.; Li, X.; Yang, H.; et al. Systematic transcriptional analysis of human cell lines for gene expression landscape and tumor representation. *Nat Commun* **2023**, *14*, 5417, doi:10.1038/s41467-023-41132-w.

Note that TRIM56 is broadly expressed in all the human cell lines listed here except the neuroblastoma cell line SH-SY5Y, which expresses negligible TRIM56 RNA.
